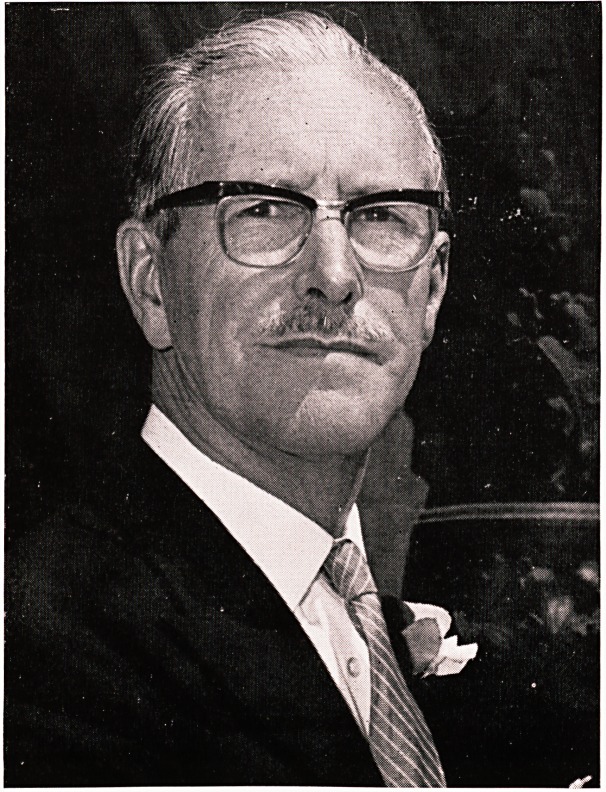# John Ridgway

**Published:** 1970-07

**Authors:** 


### Obituary Notices

### John RIDGWAY, M.B., Ch.B.

Dr. John Ridgway died on Saturday the 10th January from heart disease, which had curtailed his activities, both professional and otherwise, during the last six years of his life.

John Ridgway went to Clifton College when the family moved from Newcastle-under-Lyme to Bristol. At school his aptitudes were greatest in history and he might well have taken history at University but, fortunately for medicine, added himself to those depleted ranks of the doctors with scholastic background in the arts and classics. He was a Bristol graduate in medicine, doing his clinical work at the Bristol General Hospital. In the early thirties the clinical student was still involved wholly in one or other of the rival hospitals, the Bristol General Hospital or the Bristol Royal Infirmary. Qualifying in 1932, he did two resident appointments at the General Hospital before going as an assistant to Dr. C. Perrett and Dr. Macdonald in Kingswood and Hanham. When Dr. Perrett died in the middle thirties Dr. Ridgway acquired the branch practice at Hanham and from this he, and later his partners, developed the present practice, covering so much of Hanham and Warmley. He was always interested in anaesthesia and began to give anaesthetics for regular lists and the emergencies at Cossham Memorial Hospital, so contributing to the voluntary hospital service of that part of Bristol. The practice grew and he acquired fresh premises across the road for his surgery, showing vision both as regards the existing need and future requirements in family practice.

The outbreak of war in 1939 found him in the Royal Naval Volunteer Reserve, so that he was immediately called up and served in the navy for two years. The practice was left in the hands of locum practitioners, who proved to be too transient and unsatisfactory for the needs of this part of north-east Bristol. This led to Dr. Ridgway being released from the navy to return to his practice. He worked even harder during the war years and took on his anaesthetic commitments again at Cossham Hospital. Following the war he took his first partner, Dr. Richard W. Orton, and so commenced a partnership which to-day comprises no less than five doctors. A purpose-built extension to the surgery premises came soon after, making these one of the most advanced in Bristol for that period.

John was very fortunate in his marriage to Esmé, a nurse in the Bristol General Hospital, where the romance blossomed in his student days. They married soon after he started in general practice and she was a source of tremendous help throughout his early days, and bore the considerable brunt of keeping the practice going during his absence in the navy. They moved their home to Blenheim Road, Redland shortly after the war and here a small extension of his practice was developed. His anaesthetic commitments were extended to include sessions at Winford Orthopaedic Hospital and, shortly afterwards, his work in anaesthesia was recognised in his being appointed as one of the six general practitioner anaesthetists to augment the Anaesthetic Service in the Bristol Royal Hospital. With increasing commitments in anaesthesia, Dr. Ridgway had to give up more of his work in general practice but the partnership had already grown to four partners and it was shortly after this that Dr. and Mrs. Ridgway moved to their attractive house in Downs Park West. It was here that about seven years ago, he developed serious trouble with his heart and was altogether off work for a period of six months. As a result there had to be a major reorganisation of his life and he gave up family practice and reduced his commitments in anaesthesia. But, with these limited commitments, he seemed to be keeping in very good health until the serious setback at Christmas, from which he died two weeks later.

John was essentially a shy man, yet interested in people; very conscientious and considerate, his patients acquired great confidence in him and his practice grew rapidly. Similarly, in anaesthesia, his calm and conscientious presence at the head of the table was a great help to patient and surgeon alike.

Outside medicine, we found that he never lost his interest in history and from this never missed seeing an old church or building. He read a good deal. Music was always a favourite, particularly opera, and he made his annual visit to Glyndebourne even after his serious illness. Always a keen philatelist, he continued to add to his collection throughout his life. He was always interested in nature and particularly birds, and this took him out into the country as, apart from this, he was not interested in sports or other energetic pursuits, but greatly enjoyed walking.

John was a kindly man, a friend to his patients and his colleagues; though a sensitive personality, he was robust enough for the life of a city general practitioner. That fine upright figure, with the kindly smile, will be greatly missed by many in Bristol and we offer our sympathies to Esmé and their daughter Anna.—
A.L.E.B.

## Figures and Tables

**Figure f1:**